# SARS and Population Health Technology

**DOI:** 10.2196/jmir.5.2.e14

**Published:** 2003-06-30

**Authors:** Gunther Eysenbach

**Keywords:** Severe acute respiratory syndrome, communicable diseases, emerging, epidemiology, Internet, public health, information dissemination, Internet, computer communication networks, bioterrorism

## Abstract

The recent global outbreak of SARS (severe acute respiratory syndrome) provides an opportunity to study the use and impact of public health informatics and population health technology to detect and fight a global epidemic. Population health technology is the umbrella term for technology applications that have a population focus and the potential to improve public health. This includes the Internet, but also other technologies such as wireless devices, mobile phones, smart appliances, or smart homes. In the context of an outbreak or bioterrorism attack, such technologies may help to gather intelligence and detect diseases early, and communicate and exchange information electronically worldwide. Some of the technologies brought forward during the SARS epidemic may have been primarily motivated by marketing efforts, or were more directed towards reassuring people that "something is being done," ie, fighting an "epidemic of fear." To understand "fear epidemiology" is important because early warning systems monitoring data from a large number of people may not be able to discriminate between a biological epidemic and an epidemic of fear. The need for critical evaluation of all of these technologies is stressed.

## Introduction

Severe acute respiratory syndrome (SARS) hit the world on November 16, 2002, when the first cases of atypical pneumonia appeared in the Guangdong Province, south China. The disease quickly spread to Vietnam and Hong Kong, and from there made its way around the globe. The western hemisphere was not spared: about 37 people have died in Canada as of June 30, 2003, all in the Toronto area, and Canada remains the only region outside of Asia with deaths from SARS.

With SARS, countless media reports and company press releases promoting information and communication technology (ICT) solutions appeared (Textbox 1). Some technology firms attempted to turn lemons into lemonade by using the crisis to bring their ICT products and services into the media and to public attention. As the number of new SARS cases declines and the dust settles, it is time to ask critical questions, including which of these tools and technologies have proven useful or should be further developed and evaluated in order to be prepared for the next public health emergency.

## Role of information technology during the SARS epidemic

The public health and infectious disease research community widely praised the role of ICT in early detection as well as in fostering global collaboration and information exchange during the SARS epidemic. On March 17, 2003, WHO called upon 11 laboratories in 9 countries to join a collaborative multi-center research project on SARS diagnosis. The network took advantage of e-mail and a secure WHO Web site to share outcomes of investigations of clinical samples, electron-microscope pictures of viruses, sequences of genetic material for virus identification and characterization, and postmortem tissues from SARS cases in real time [1]. Individual departments of affected hospitals also used Web sites and e-mail to rapidly disseminate clinical findings to health professionals [2]. "This is a form of early warning and communication that would not have been possible if the SARS Virus had appeared ten years ago," notes Dr. Kimball of the APEC Emerging Infections Network [3], and Julie Gerberding, director of the US Centers for Disease Control and Prevention, writes in an editorial in the New England Journal of Medicine that "use of the Internet has sped information exchange and helped overcome the problems presented by asynchrony in the activities of investigators in many time zones" [4].

Journal editors celebrated themselves and the Internet for being able to publish articles about SARS at the speed of electrons [5]. However, the role of journals — even if with electronic preprint versions and fast track peer-review — dwarfs compared with the role of the Internet in information dissemination of SARS. As of June 30, 2003, PubMed lists 881 articles containing the search words ["severe acute respiratory syndrome" OR SARS]. In contrast, Google finds 358000 pages with the phrase "severe acute respiratory syndrome" (not counting non-English pages or pages which contain only the abbreviations SARS or SRAS for "syndrome respiratoire aigu sévère").

The World Health Organization (WHO) also praised the role of GPHIN (Global Public Health Intelligence Network) for early detection of SARS, claiming that "GPHIN provided some of the earliest alerts to the November outbreak in China" [6]. GPHIN is part of WHOs Global Outbreak Alert and Response Network [7]; it was developed and is operated by Health Canada's Centre for Emergency Preparedness and Response. It is essentially an Internet crawler specialized in detecting news articles indicating unusual events relevant to public health: GPHIN continually scans more than 400 international sources for news of any outbreaks of 31 communicable diseases, as well as articles about natural disasters and drug-resistant pathogens, rather than relying on "official" reports from government sources (which may be reluctant to report disease outbreaks to avoid economic disruptions). The approach is obviously insufficient when disease outbreaks occur in developing countries where little information finds its way into news media and the Internet, or in countries where the media a controlled by the government. GPHIN seems to be stretched to its limits (when the author requested access to the system the reply was "we have now reached our limit on the number of users that can have access to the system"). GPHIN is currently being upgraded to include more languages, including Chinese (which during the SARS crisis was not yet implemented).

## Population Health Technology

eHealth, consumer health informatics, and public health informatics are emerging fields that have a clear public health aspect, in that they include technologies that can be used to improve the health of entire populations, not just individuals. *Population health technology* is a recent umbrella term subsuming applications of technologies such as the Internet, wireless devices, mobile phones, smart appliances, or smart homes (domotics) that have a population focus and the potential to improve public health. In principle, all sorts of home-monitoring devices, from digital fever thermometers to asthma-monitoring devices, could be modified to function as early detection systems, ie, to transmit data — wirelessly or through the Internet — to central data-mining facilities, which may detect emerging patterns indicating disease outbreaks. Among the challenges of all of these systems are ethical and privacy concerns — it is a difficult balance between gathering data from thousands of people with being able to track down infected individuals on the one hand, and protecting the privacy of people on the other hand. Hospitals and pharmacies of tomorrow may also feed data into such central data-mining systems. In addition, there may be a role for detecting patterns of information and communication flows on the Internet. At the Centre for Global eHealth Innovation we have been experimenting with monitoring search requests people enter into search engines, to evaluate whether it is possible to detect increases or changes in health-related requests using automatic methods [8]. The sensitivity of such methods for detecting disease outbreaks or bioterrorism attacks remains to be evaluated — in our search term experiment it did not seem to be sensitive enough in the case of SARS.

**Examples for eHealth solutions offered during the SARS crisis**

**Figure 1:**
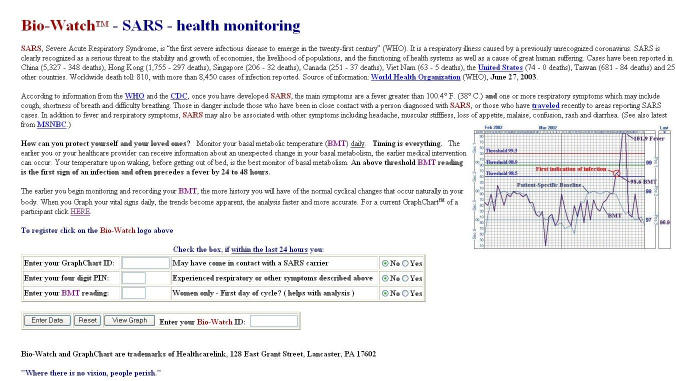
Healthcarelink

**Figure 2:**
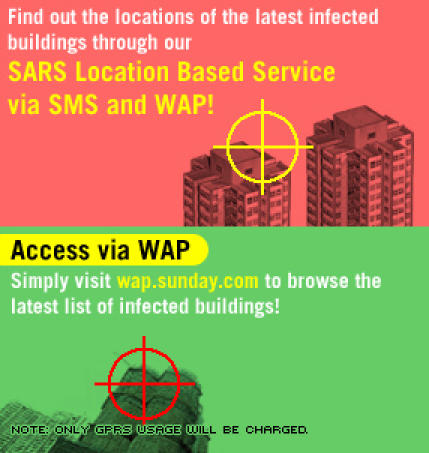
Advertisements of Sunday Communications in Hong Kong

- Healthcarelink (http://www.healthcarelink.md) has developed a monitoring program that claims to detect severe acute respiratory syndrome before symptoms occur and which — by aggregating data from a large number of patients — also promises to detect bioterror outbreaks. Patients take their temperature daily in the morning and report the results by phone, fax, or Internet. The company publishes the graphs on the Internet for patients and physicians to review. The data, along with information on a person's travel history, can alert health workers to potential SARS patients and bioterrorist attacks (Figure 1). One of the open questions is, of course, how to motivate a large number of people to measure their temperature daily and to voluntarily enter this information into a Web form.
- A very similar approach is behind the idea of Swedish company MedDay (http://www.medday.com), which proposes that people enter symptoms into PDAs or smart phones, which would wirelessly transmit the information to a health or infectious-disease center, which could aggregate and monitor these data. The company claims that the system can be used as an early warning system for a nationwide outbreak of infectious diseases, chemical attack, or other disease. The company is surfing the bioterrorism wave as it simply rebranded its PharmaPoint software, originally developed for remote patient monitoring by physicians, into RegPoint, hoping that it will be used by governments to keep track of the health of populations. There are open questions about the sensitivity of the system to detect outbreaks against a "background noise" as well as about privacy issues.
- Sunday Communications, a Hong Kong mobile phone operator, launched a mobile phone service that promised to alert subscribers if they are near infected buildings. Those opting for the service had their phones tracked, and would be warned by SMS (short message service) whenever they strayed within a kilometer of a building where there had been instances of SARS infections. It is unknown whether this system prevented a single new SARS case (Figure 2).
- In Singapore, health officials tested electronic tracking systems that monitor the movements of every person who enters a public hospital. Staff and visitors wear credit card-sized RFID (radio frequency identification) tags around their neck to communicate their location to sensors hidden in the hospital ceilings, thereby enabling officials to track all encounters with other persons. Hospitals will save movement records for 20 days — twice the incubation period for SARS. If one person turns out to be infected, the database allows rapid identification of all encounters — health officials say it is 10 times faster than traditional methods of asking infected people whom they had contact with.

## Information Technology and the Epidemiology of Fear

In the last week of June, Ontario presented Ottawa with a breakdown of $945 million (Canadian) in SARS-related costs. Those include $395 million for hospitals and health-care institutions for extra staff expenses, protective gear, clinics, and isolation rooms. Another $330 million went to replace lost wages for quarantined health-care workers. Even more serious may be the consequences of the SARS-related "epidemic of fear" [9]. Millions of dollars were lost due to missed business — tourists and business travelers staying away from the hot spots. Worse, as in this particular incident hospitals were the hubs of the outbreaks, patients postponed or delayed important hospital visits. It is difficult to estimate how many patients have been harmed by avoiding hospitals — at least outside of China these may be more than those actually killed by the disease. Kelly MacDonald, a University of Toronto infectious-disease expert, estimates that four times as many Ontarians will die from lack of medical attention caused by the SARS outbreak as will die from the disease itself [10]. To understand the epidemiology of fear in the context of population health technology is important for at least two reasons.

First, some ICT applications that have been advocated sometimes played a role as a psychological "duct tape of the war against fear." Indeed, Bruce Hicks, group managing director of Sunday Communications, the operator that launched the mobile phone service that alerts subscribers when they are near infected buildings (see above), was quoted as describing his service with these words: "With the dial of a few digits, subscribers can quickly get the peace of mind they need to go about their everyday lives." — speaking to the fact that it is not primarily the spread of SARS but the fear that is addressed by this service. Thermal-imaging scanners set up at airports to screen travelers for signs of SARS may have had a similar role: to assure people that "something is being done," and to prevent economic damages. In fact, there is limited evidence on the sensitivity and specificity of this technology to identify passengers with fever. Information and communication technology can also help to keep the health care system accessible in cases of disease (or fear) outbreaks. For example, Singapore General Hospital, introduced during the SARS crisis an online physiotherapy program allowing physical therapists to remotely monitor patients in their homes. Using a webcam, patients can communicate with their therapists, who can in turn show their patients new exercises and give them feedback on their progress [11].

The second reason why we need to understand the epidemiology of fear in the context of population health technology is that some technologies used as early warning system may not be able to discriminate between a true biological epidemic and an epidemic of fear. This is especially true for systems relying on users entering symptoms, systems designed to detect changes in patterns of health care utilization or other databases, or systems analyzing information and communication patterns on the Internet or in news media. Such early-warning systems may pick up changes in collective behavior triggered by a mere epidemic of fear. For example, thousands of New Yorkers buying duct tape did not indicate a bioterrorism attack, but a fear epidemic. Similarly, runs on doctors or pharmacies may either indicate mass hysteria (a highly-prevalent phenomenon in our society), or a bioterrorism attack (a far less prevalent event). The predictive value of such early warning systems is thus inherently low. False positive warnings lead to media reports, and lead to further changes in the public's behavior — a potentially-devastating positive-feedback loop.

## Conclusions

The recent SARS outbreak provides an opportunity to analyze and study the use of population health technology, and to learn lessons for future public health emergencies, including acts of bioterrorism. Most importantly, it should be a stimulus to critically evaluate these technologies and to provide directions for further research and development. Population health technology clearly has a vast potential to increase our preparedness for the next public-health emergency, but it also raises many questions related to ethics, libertarian values, and privacy, and has the potential to fuel an epidemic of fear and collective mass hysteria.
